# The Impact of Surgery duration and Surgery End Time on Postoperative Sleep in Older Adults

**DOI:** 10.23937/2572-4053.1510034

**Published:** 2021-08-16

**Authors:** Danielle Tran, Christopher Tang, Sanam Tabatabai, Devon Pleasants, Christopher Choukalas, Jie Min, Quyen Do, Laura Sands, Kathryn Lee, Jacqueline M Leung

**Affiliations:** 1 Department of Anesthesia and Perioperative Care, University of California San Francisco, USA; 2 Virginia Tech, Department of Statistics, USA; 3 Virginia Tech, Center for Gerontology, USA; 4 School of Nursing, University of California San Francisco, USA

**Keywords:** Actigraphy, Postoperative sleep disruption, Surgery duration, Surgery end time, Wake after sleep onset, Daytime inactivity, Older adults

## Abstract

**Objectives/Background:**

Sleep disruption is prevalent in older patients. No previous studies have considered the impact of surgery duration or surgery end time of day on postoperative sleep disruption. Accordingly, we examined the duration of surgery and surgery end times for associations with postoperative sleep disruption.

**Methods:**

Inclusion criteria were patients ≥ 65 years of age undergoing major, non-cardiac surgery. Sleep disruption was measured by wrist actigraphy and defined as wake after sleep onset (WASO) during the night, or inactivity/sleep time during the day. The sleep opportunity window was set from 22:00 to 06:00 which coincided with “lights off and on” in the hospital. WASO during this 8-hour period on the first postoperative day was categorized into one of three groups: ≤ 15%, 15–25%, and > 25%. Daytime sleep (inactivity) during the first postoperative day was categorized as ≤ 20%, 20–40%, and > 40%. Statistical analyses were conducted to test for associations between surgery duration, surgery end time and sleep disruption on the first postoperative day and following night.

**Results:**

For this sample of 156 patients, surgery duration ≥ 6 hours and surgery end time after 19:00 were not associated with WASO groups (p = 0.17, p = 0.94, respectively). Furthermore, daytime sleep was also not affected by surgery duration or surgery end time (p = 0.07, p = 0.06 respectively).

**Conclusion:**

Our hypothesis that patients with longer duration or later-ending operations have increased postoperative sleep disruption was not supported. Our results suggest the pathophysiology of postoperative sleep disruption needs further investigation.

## Introduction

Although all the functions of sleep are still unknown, sleep is necessary to maintain our overall health [1]. Wakefulness in the daytime and sleep at night are normally expected, but humans can experience a bimodal sleep pattern, with the major peak during the night and a smaller peak or nap in the afternoon [2]. Normally, adults sleep 7 to 9 hours, awaken 2 to 6 times during the night, and spendless than 10% of total sleep time awake after initial sleep onset [3]. However, sleep patterns also change with aging, resulting in more wake time and lower sleep efficiency [1].

Sleep disruption can affect vital regulatory processes such as metabolic and immune function [4], resulting in slower postoperative recovery, particularly in vulnerable older surgical patients. Sleep disruption may be the result of a variety of factors, including pain and hospital environment-related factors such as noise and light exposure from procedures or intensive monitoring during the night [5].

Furthermore, sleep disruption can affect postoperative outcomes. Our prior study demonstrated that preoperative sleep disruption measured by wrist actigraphy was associated with postoperative delirium [6]. Another study of 110 patients undergoing unilateral total knee replacement surgery showed that patients who had more sleep disruptions and greater pain levels during the first month after surgery reported more functional limitations three months after surgery, affecting recovery [7]. Therefore, exploring factors that contribute to postoperative sleep disruption in older patients is a crucial first step toward implementing effective strategies to promote a more rapid recovery and better health outcomes.

Past studies have examined the impact of hospital environment [5], type of surgery [8], and mode of anesthesia [9] on postoperative sleep disruption. Specifically, patients’ pain levels and noise from neighboring patients and nursing staff [5] have been cited as one of the factors contributing to postoperative sleep disturbance. However, no research has yet examined the impact of surgery duration orend time on postoperative sleep-in older patients. Specifically, long and/or late-ending surgeries may alter the patient’s circadian rhythms and disrupt postoperative sleep or daytime activity. Accordingly, our study aimed to examine the effects of surgery durationand end time of day on postoperative sleep during the first postoperative day and following night. We hypothesized that patients with longer or later-ending surgeries would have more wake time during the night of the first postoperative day and more daytime inactivity on the first postoperative day compared to patients with shorter or earlier completion of surgery.

## Materials and Methods

### Study design

This is a secondary analysis of a prospective cohort study conducted at the University of California, San Francisco Medical Center and San Francisco Veteran Affairs Medical Center investigating the association between sleep and postoperative delirium in older adults. The study received IRB approval and all participants provided written and informed consent.

### Inclusion/exclusion criteria

The study recruited patients aged ≥ 65 years, undergoing major elective non-cardiac surgery with general anesthesia who were also fluent in English, had an anticipated hospital length of stay of two nights or longer, and able to understand and consent for the study. Exclusion criteria included a history of restless leg syndrome, periodic limb movement disorder, or obstructive sleep apnea.

### Data collection

**Figure 1:**
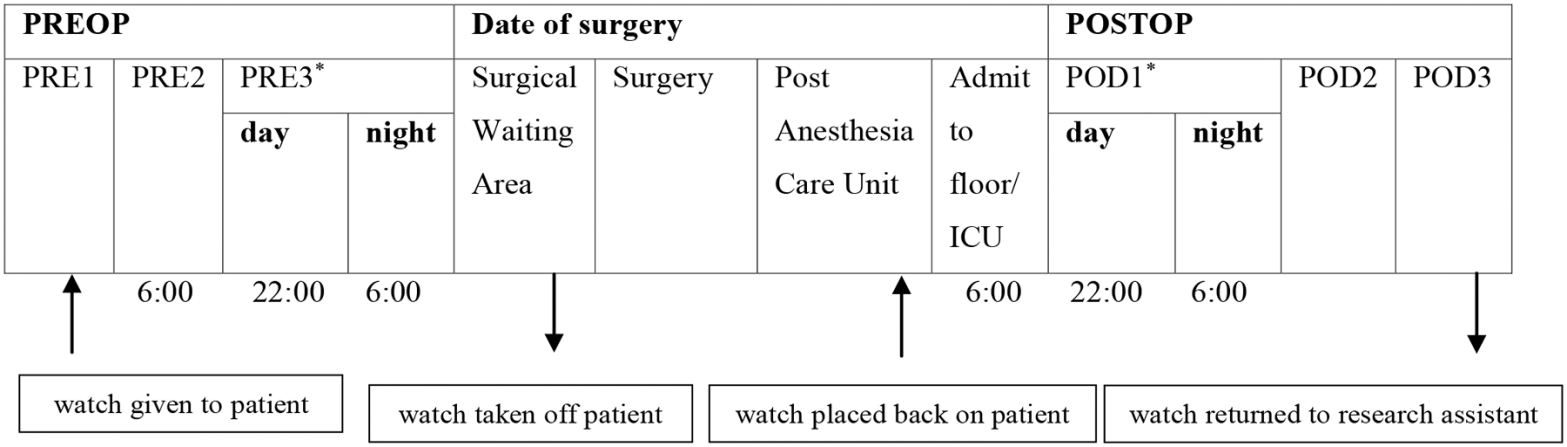
Timeline of events. *: Days of interest PRE: Preoperative Day; ICU: Intensive Care Unit; POD: Postoperative Day Sleep was examined on the day and night closest to the date of surgery, and on the day and night of the first postoperative day. Note: Surgery start and end times varied for each patient. For patients with late surgery end times, the research assistant gave the watch to the nurse in the post anesthesia care unit, who would then place the watch back on the patient.

To assess sleep and wake time, patients received the Philips Actiwatch 2 (Philips Respironics, Murrysville, Pennsylvania) in person or by mail and were instructed to wear the actigraph for at least two days and nights before their surgery date. The actigraph was removed beforethe patient went into the operating room and placed back on the patient after surgery before discharge from the post anesthesia care unit. Starting from the night immediately after surgery, patients wore the actigraph for at least two days and nights postoperatively, after which the actigraph was retrieved by a trained research assistant. The timeline of when the actigraph was worn and the periods included in the analysis is shown in Figure 1.

Data from the wrist actigraph were uploaded for analyses onto the Philips Respironics Actiware Software (Version 6.0.9, 2020). Actigraphy data were dichotomized as night (22:00 to 06:00) and day (06:00 to 22:00) to reflect the hospital’s routine practice with “lights out” at 22:00 and vital sign assessments beginning at 06:00 for elective surgical patients. To obtain actigraphy values, trained research assistants used the auto scoring default algorithm provided by the Actiware software (Version 6.0.9, 2020) to minimize any researcher sleep scoring bias.

### Measures

#### Nighttime sleep:

Rest intervals were set from 22:00 to 6:00 every night for each patient, from which the Actiware software’s (Version 6.0.9, 2020) algorithm generated sleep intervals and numerical sleep output values. Actigraphy values for night time sleep were obtained from the Actiware software’s sleep interval. Sleep disruption was estimated with actigraphy values for the percentage of wake after sleep onset (WASO) between 22:00 and 06:00 during the night of the first postoperative day. WASO was categorized into three groups based on the Pittsburgh Sleep Quality Index scoring algorithm [10]: ≤ 15%, 15–25%, and > 25%. WASO ≤ 15% indicates minimally disrupted sleep, and > 25% indicates moderately to highly disrupted sleep.

#### Daytime inactivity/sleep:

Daytime inactivity recorded by the actigraph was used as a measure to estimate daytime sleep on the first postoperative day from 6:00 to 22:00. Daytime inactivity in the Actiware software, designated in the active interval as sleep time, reflects the total number of epochs scored as sleep or inactive from 6:00 to 22:00. To evaluate the potential impact of surgery duration and end time of day on daytime inactivity/sleep, the percentage of sleep time or inactivity from 06:00 to 22:00 on the first postoperative dayprovided by the Actiware software was examined. Percentage of daytime inactivity was divided into tertiles based on daytime inactivity measured pre-operatively in our patient cohort: ≤ 20%, 20–40%, and > 40%, based on their pre-operative daytime inactivity.

### Actigraphy analysis settings

The analysis settings were set at medium wake threshold and ten minutes of immobility for sleep onset and offset, with epoch lengths of either 30 seconds or 60 seconds. Consecutive epochs of inactivity for two or more hours during the daytime were excluded under the assumption that the watch was not being worn. Trained research assistants also visually examined the actograms to ensure the software’s algorithm properly captured sleep. Upon visual examination, there were seven cases where the software algorithm did not score sleep for multiple consecutive nights when it was evident the patient was sleeping. For these cases, analysis settings were adjusted in different combinations, such as high or automatic wake threshold and one or five minutes of immobility for sleep onset and offset, for the software to score sleep. Of these seven cases, sleep was not captured for three cases despite adjusting the analysis settings. There was also one patient who slept on all other nights except the night prior to surgery. In total, for the four patients who did not sleep the night before or after surgery, %WASO was imputed to 100%. A sensitivity analysis was done excluding these four patients to determine if results would differ.

### Demographics, pre-operative sleep, and surgical characteristics

The patient’s medical record was used to obtain demographic characteristics of age, gender and race, as well as surgery characteristics that included type (general, major joint and spine), surgery end time (after 19:00), surgery duration (≥ 6 hours). Admissions to the intensive care unit on the first postoperative day were also noted. Post-operative pain (≤ 5) was self-reported by the patient during an in-person assessment on the first postoperative day. Pre-operative WASO was calculated from the software as described above forthe night closest to the surgery date between 22:00 to 6:00. Similarly, pre-operative daytime inactivity/sleep during the day closest to the surgery date between 6:00 to 22:00 was calculated as described above.

### Statistical analysis

Frequencies and percentages were used to describe the sample’s demographic, surgical, and sleep characteristics. Chi-square tests were computed to assess the association between demographic, pre-operative sleep, and surgical characteristics associated with the sleep disruption variables. Fisher’s exact test was used in cases of expected cell frequencies < 5. All analyses were conducted in R version 4.0.4. Statistical significance was set at p < 0.05.

## Results

**Figure 2:**
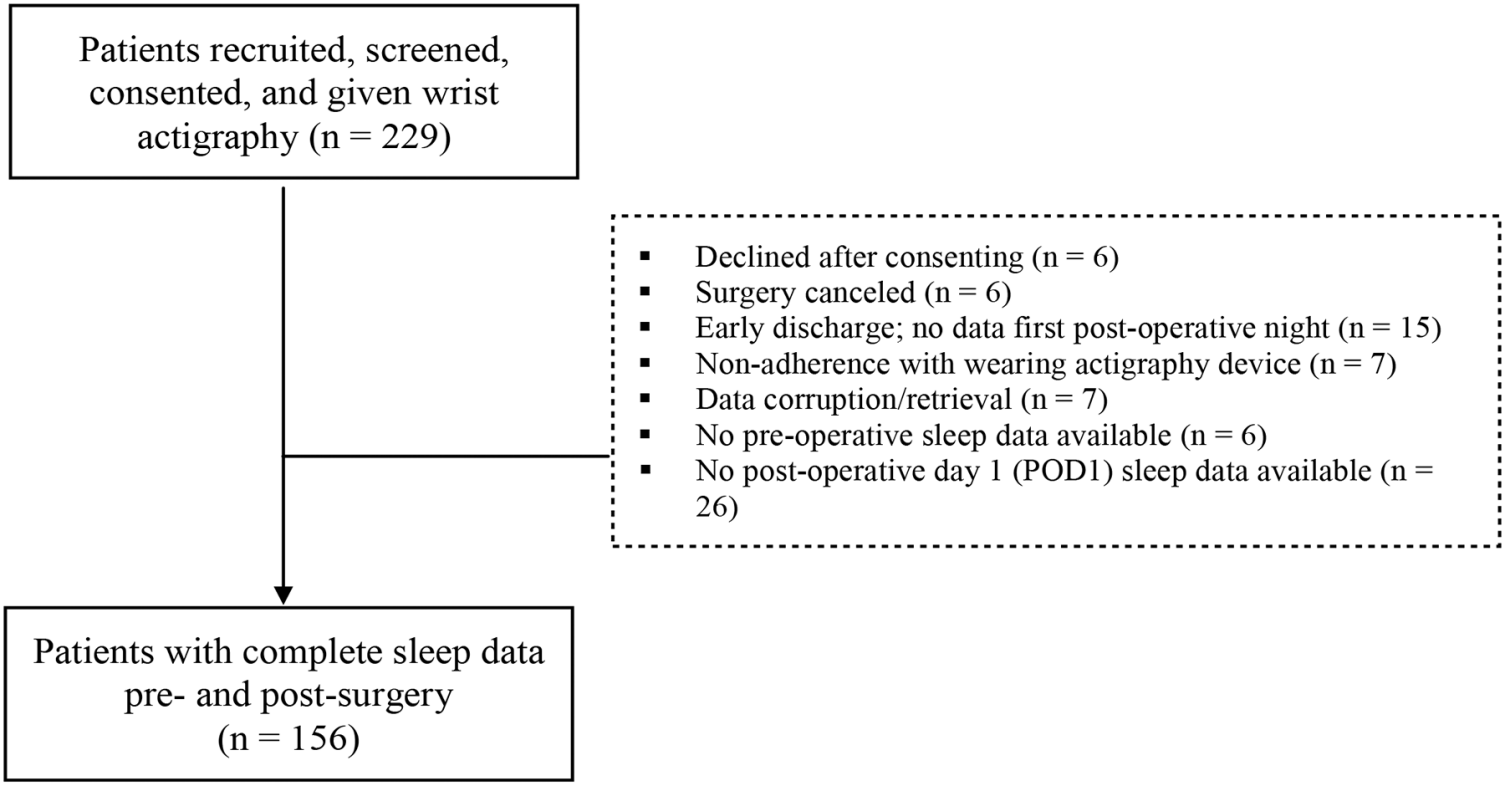
Study participant’s flow chart. Of 229 patients initially recruited, 73 were excluded due to later declining to participate, surgery cancellation, early discharge, nonadherence with wearing the actigraph, data corruption, or no available preoperative or postoperative sleep data. Our study has a total sample size of 156 patients.

Of 229 patients who consented to participate in the study, 73 patients were excluded for a variety of reasons (Figure 2), including surgery cancellation, early discharge, non-adherence with wearing the actigraph, or data corruption (Figure 2). Therefore, participants for this analysis included 156 elective surgical patients recruited as part of the parent study to determine postoperative delirium in older adults.

The patients’ demographic and surgical characteristics are shown in Table 1. Two-thirds of patients were 75 years of age or older, 54% were male, and 76% self-identified as Caucasian. General surgery was the most common surgery type (49%) followed by spine surgery (36%) and joint arthroplasty (15%). One-third of patients had surgical durations of 6 hours or longer; most surgical procedures ended before 19:00 while 11% ended after. About 70% of patients reported having a pain level > 5 after surgery. Only 4% were admitted to intensive care postoperatively.

Before surgery, 54% of patients had ≤ 15% WASO and only 17% experienced moderately or highly interrupted night sleep (> 25% WASO). Postoperative WASO was similar, with 60% of patients experiencing ≤ 15% WASO and 16% of the patients experiencing > 25% WASO. Preoperatively, only 21% of the patients spent > 40% of the day inactive or sleeping, compared to the first postoperative day when the majority (65%) of patients spent > 40% of the day inactive or sleeping.

Table 2 shows patient and surgery characteristics stratified by WASO sleep disruption group on the night of the first postoperative day. Surgery duration and end time were not associated with WASO. Age, gender, race, postoperative pain level, intensive care unitadmission, surgery type, and preoperative %WASO were not significantly associated with postoperative WASO.

Table 3 shows the patient and surgery characteristics by daytime inactivity/sleep. Age, gender, race, postoperative intensive care unit admission and postoperative pain level were not related to inactivity/sleep on the first postoperative day. Postoperative daytime inactivity was significantly different across types of surgery type. Patients with major joint surgery were the most active while patients who underwent general surgery spent more than 40% of the day inactive or sleeping. Surgery end time and surgery duration were not significantly associated with levels of daytime sleep, though there was a trend indicating that patients with a surgery end time after 19:00 had less interrupted sleep at night, and patients with surgery duration ≥ 6 hours had more interrupted sleep. A sensitivity analysis was performed using data that excluded the four patients who did not sleep either on the preoperative or postoperative night. While the analysis results did not change substantially, the association between surgery duration and daytime inactivity improved from p = 0.07 to p = 0.03.

## Discussion

In this study, postoperative sleep disruption was common as shown by many awakenings at night, but similar to our prior findings [6], preoperative sleep disruption was also prevalent in this cohort, confirming similar observations from our previous study. Possible explanations for disrupted sleep may include pain, anxiety, or other more chronic symptoms such as depression or insomnia. Our results show that surgery duration and surgery end time was not associated with postoperative sleep disruptionon the night of the first postoperative day.

Our results cannot be directly compared to findings from previous studies as none had examined the potential impact of surgery duration or surgery end time on postoperative sleep disruption. Our hypotheses that longer surgery or late surgery end times would lead to a shift in the sleep-wake cycle, resulting in more wake time during the night, were not supported. The lack of association may be in part due to the findings that our cohort had substantial sleep disruption even before surgery as measured by wake after sleep onset and daytime sleep. This trend continues after surgery, which likely explains why a significant increase in the amount of sleep disruption from before to after surgery was not seen.

We also observed that a fifth of the patients had substantial daytime inactivity/sleep prior to surgery. The etiology of this extensive daytime inactivity is unclear, but these were patients awaiting major elective surgery and many might have been in pain, with reduced ambulation resulting in inactivity measured by actigraphy rather than true sleep. After surgery, patients overall had an increase in daytime sleep, but this was not associated with surgery duration or end time. The lack of association may be because patients are generally more inactive after surgery due to pain, sedation, or frequent need of invasive monitoring. Thus, other factors may be responsible for the observed increase in daytime sleep postoperatively and should be explored in future studies.

While an extensive review of literature reveals postoperative sleep disruption may be due to factors such as pain [11], noise level [5,12], and nursing activities [13,14], none of these studies have addressed the possible influence of surgery end time. For studies that included the effect of surgery times, there was an association between later surgery start times and postoperative complications [15] or adverse events [16,17]. Although we did not find an association between surgery end time and postoperative sleep disruption, our study is novel in that we examined the effects of surgery end timing on postoperative sleep which has not been previously reported.

A few studies have alluded to the effects of surgery on postoperative sleep. Our findings regarding type of surgery and surgery duration differ from a Danish study in which open abdominal surgery was compared to the typically shorter laparoscopic cholecystectomy using a self-reported sleep quality measure [8]. They found no significant difference in night sleep by type of surgery, but the open abdominal surgery group of 15 patients reported more postoperative daytime sleep compared to the 12 patients in the laparoscopic group. Our results show that daytime inactivity differs by types of surgery. We cannot discern the reasons for this difference but postoperative pain, recovery, and readiness to participate in physical therapy and ambulation, and other patient related factors may contribute to differences in daytime inactivity across different surgery types.

Although the pathophysiology on postoperative sleep disruption has yet to be fully elucidated, researchers have examined postoperative changes in core body temperature and in melatonin and cortisol hormones known to be involved in sleep/wake circadian rhythms. In astudy of 11 patients undergoing major abdominal surgery, there was delay in the timing of melatonin on the first postoperative day and temperature rhythms on the second postoperative day, as well as a change in cortisol and melatonin secretion [18]. Surgery duration was also positively correlated with the delayed melatonin rhythm [18]. Because melatonin, cortisol, and core body temperature levels were not assessed in our sample, changes in these circadian biomarkers should be examined in future studies.

There are some limitations in our study to consider. First, the Philips Actiwatch 2 does not have an on-wrist/off-wrist detection feature to automatically exclude periods when the device is removed from the wrist. Therefore, we may have overestimated daytime inactivity. However, we manually inspected each actogram and excluded periods in which the actigraph was likely not on the patient’s wrist, as indicated by 2 hours or more continuous epochs of inactivity and using the light level as a general guide. Additionally, wrist actigraph monitoring is based on movement and is unable to distinguish between sleep and lack of movement or immobilization due to such factors as pain. Thus, daytime inactivity is not a precise representation for daytime sleep, but compared to other studies with self-report measures, our Actigraphy measure allowed for a consistent estimate of daytime sleep across all patients in our cohort.

Despite these limitations, we found that surgery duration and surgery end time of day had no effect on postoperative nighttime sleep in this sample of older adults. While many studies have focused on the effects of environmental factors that contribute to sleep disruption in the hospital unit, examining the effects of surgery duration and end time presents a new approach. Our findings demonstrate the need for more research on potential modifiable risk factors for postoperative sleep disruption.

Overall, in this cohort of older patients undergoing major noncardiac surgery, surgery duration and surgery end time were unrelated to postoperative sleep disruption at night or inactivity during the day. However, surgery type was associated with daytime inactivity on the first day after surgery while still hospitalized. Future studies should identify other causes of postoperative sleep disruption.

## Figures and Tables

**Table 1:** Patient demographics and surgery characteristics.

Patient Characteristics (N = 156)	
Age (% 75+)	67%
Gender (% Male)	54%
Race (% White)	76%
**Surgery Characteristics**	
Surgery type	
General (%)	49%
Spine (%)	36%
Major joint arthroplasty (%)	15%
Surgery duration (% 6 hours or more)	32%
Surgery end time (% 19:00 and later)	11%
**Postoperative Events**	
Postoperative Pain(% ≤ 5)	31%
Admission to Intensive Care Unit (%)	4%
**Sleep Characteristics**	
Preoperative % WASO	
≤ 15%	54%
15–25%	29%
> 25%	17%
Preoperative % day sleep	
≤ 20%	22%
20 to 40%	57%
> 40%	21%
Postoperative % day sleep	
≤ 20%	7%
20 to 40%	28%
> 40%	65%
Postoperative % WASO	
≤ 15%	60%
15–25%	24%
> 25%	16%

WASO: Wake After Sleep Onset.

This table illustrates the demographics, surgery, and sleep characteristics of our sample.

**Table 2:** Univariate associations between level of sleep interruption during the night of POD1 and patient and surgery characteristics.

	Least interrupted WASO ≤ 15%	Somewhat interrupted WASO 15–25%	Moderately/highly interrupted WASO > 25%	
	(N = 94)	(N = 37)	(N = 25)	p-value
**Patient Characteristics**				
Age, % 75+ years	66%	62%	80%	0.31
Gender, % male	51%	62%	52%	0.51
Race, % White	73%	81%	76%	0.65
**Surgery Characteristics**				
Surgery type, %				
General	54%	41%	40%	
Major joint	15%	19%	12%	0.42*
Spine	31%	41%	48%	
Surgery End Time, % 19:00 and later	11%	4%	2%	0.94*
Surgery Duration, % 6 hours or more	30%	27%	48%	0.17
**Postoperative Events**				
Postoperative pain, % ≤ 5	33%	28%	25%	0.68
ICU, % admitted	5%	3%	0%	0.72*
**Sleep Characteristics**				
Preoperative WASO				0.65*
≤ 15%	54%	62%	44%	
15 to 25%	28%	27%	36%	
> 25%	18%	11%	20%	
Preoperative daytime sleep				0.64
≤ 20%	24%	22%	16%	
20 to 40%	53%	62%	68%	
> 40%	24%	16%	16%	

* Results from Fisher’s exact test

WASO: Wake After Sleep Onset; ICU: Intensive Care Unit; POD1: Post-Operative Day 1

WASO groupings are based on previously defined categories [10].

**Table 3:** Univariate associations between level of daytime sleep on POD1 and patient and surgery characteristics.

	Daytime inactivity/sleep ≤ 20%	Daytime inactivity/sleep 20% to 40%	Daytime inactivity/sleep > 40%	
	(N = 11)	(N = 43)	(N = 102)	p-value
**Patient Characteristics**				
Age, % 75+ years	64%	58%	72%	0.30*
Gender, % male	64%	56%	52%	0.73
Race, % White	55%	86%	74%	0.07*
**Surgery Characteristics**				
Surgery type, %				
General	27%	35%	57%	
Major joint	45%	21%	10%	**0.01** *
Spine	27%	44%	33%	
Surgery End Time, % 19:00 and later	18%	2%	14%	0.06*
Surgery Duration, % 6 hours or more	27%	18%	38%	0.07*
**Postoperative Events**				
Postoperative pain, % ≤ 5	27%	35%	29%	0.77*
ICU, % admitted	0%	0%	6%	0.27*
**Sleep Characteristics**				
Preoperative WASO				0.25*
≤ 15%	45%	51%	57%	
15 to 25%	27%	40%	25%	
> 25%	27%	9%	19%	
Preoperative daytime sleep				0.41*
≤ 20%	36%	28%	18%	
20 to 40%	55%	56%	58%	
> 40%	9%	16%	24%	

* Results from Fisher’s exact test

WASO: Wake After Sleep Onset; ICU: Intensive Care Unit; POD1: Postoperative Day 1

Daytime inactivity groupings are based on the frequency distribution of daytime inactivity for our cohort.

## References

[1] Colten HR, Altevogt BM (2006) Sleep physiology. In: Sleep disorders and sleep deprivation: An unmet public health problem. National Academies Press, US. 20669438

[2] Bes F, Jobert M, Schulz H (2009) Modeling napping, post-lunch dip, and other variations in human sleep propensity. Sleep 32: 392–398. 19294959 10.1093/sleep/32.3.392 PMC2647793

[3] Berger AM, Parker KP, Young-McCaughan S, Mallory GA, Barsevick AM, (2005) Sleep wake disturbances in people with cancer and their caregivers: State of the science. Oncol Nurs Forum 32: E98–E126. 16270104 10.1188/05.ONF.E98-E126

[4] Besedovsky L, Lange T, Born J (2012) Sleep and immune function. Pflügers Arch J Physiol 463: 121–137. 22071480 10.1007/s00424-011-1044-0 PMC3256323

[5] Dolan R, Huh J, Tiwari N, Sproat T, Camilleri-Brennan J (2016) A prospective analysis of sleep deprivation and disturbance in surgical patients. Ann Med Surg 6: 1–5. 10.1016/j.amsu.2015.12.046 PMC4735557 26909151

[6] Leung JM, Sands LP, Newman S, Meckler G, Xie Y, (2015) Preoperative sleep disruption and postoperative delirium. J Clin Sleep Med 11: 907–913. 25979094 10.5664/jcsm.4944 PMC4513268

[7] Cremeans-Smith JK, Millington K, Sledjeski E, Greene K, Delahanty DL (2006) Sleep disruptions mediate the relationship between early postoperative pain and later functioning following total knee replacement surgery. J Behav Med 29: 215–222. 16496209 10.1007/s10865-005-9045-0

[8] Gögenur I, Rosenberg-Adamsen S, Kiil C, Kjaersgaard M, Kehlet H, (2001) Laparoscopic cholecystectomy causes less sleep disturbance than open abdominal surgery. Surg Endosc 15: 1452–1455. 11965464 10.1007/s004640090086

[9] Kjølhede P, Langström P, Nilsson P, Wodlin NB, Nilsson L (2012) The impact of quality of sleep on recovery from fast-track abdominal hysterectomy. J Clin Sleep Med 8: 395–402. 22893770 10.5664/jcsm.2032 PMC3407258

[10] Buysse DJ, Reynolds CF 3rd, Monk TH, Berman SR, Kupfer DJ (1989) The Pittsburgh Sleep Quality Index: A new instrument for psychiatric practice and research. Psychiatry Res 28: 193–213. 2748771 10.1016/0165-1781(89)90047-4

[11] Miller A, Roth T, Roehrs T, Yaremchuk K (2015) Correlation between sleep disruption on postoperative pain. Otolaryngol Head Neck Surg 152: 964–968. 25715354 10.1177/0194599815572127

[12] Christensen M (2005) Noise levels in a general surgical ward: A descriptive study. J Clin Nurs 14: 156–164. 15669924 10.1111/j.1365-2702.2004.01040.x

[13] Çelik S, Öztekin D, Akyolcu N, İşsever H (2005) Sleep disturbance: The patient care activities applied at the night shift in the intensive care unit. J Clin Nurs 14: 102–106. 15656854 10.1111/j.1365-2702.2004.01010.x

[14] Le A, Friese RS, Hsu C-H, Wynne JL, Rhee P, (2012) Sleep disruptions and nocturnal nursing interactions in the intensive care unit. J Surg Res 177: 310–314. 22683076 10.1016/j.jss.2012.05.038

[15] Kelz RR, Freeman KM, Hosokawa PW, Asch DA, Spitz FR, (2008) Time of day is associated with postoperative morbidity: An analysis of the national surgical quality improvement program data. Ann Surg 247: 544–552. 18376202 10.1097/SLA.0b013e31815d7434

[16] Wright MC, Phillips-Bute B, Mark JB, Stafford-Smith M, Grichnik KP, (2006) Time of day effects on the incidence of anesthetic adverse events. Qual Saf Health Care 15: 258–263. 16885250 10.1136/qshc.2005.017566 PMC2564010

[17] Cortegiani A, Gregoretti C, Neto AS, Hemmes SNT, Ball L, (2019) Association between night-time surgery and occurrence of intraoperative adverse events and postoperative pulmonary complications. Br J Anaesth 122: 361–369. 30770054 10.1016/j.bja.2018.10.063

[18] Gögenur I, Ocak U, Altunpinar Ö, Middleton B, Skene DJ, (2007) Disturbances in melatonin, cortisol and core body temperature rhythms after major surgery. World J Surg 31: 290–298. 17180564 10.1007/s00268-006-0256-5

